# Marine Carbohydrate-Based Compounds with Medicinal Properties

**DOI:** 10.3390/md16070233

**Published:** 2018-07-09

**Authors:** Ariana A. Vasconcelos, Vitor H. Pomin

**Affiliations:** 1 Program of Glycobiology, Institute of Medical Biochemistry Leopoldo de Meis, and University Hospital Clementino Fraga Filho, Federal University of Rio de Janeiro, Rio de Janeiro, RJ 21941-913, Brazil; arianaavasconcelos@gmail.com; 2 Department of BioMolecular Sciences, Division of Pharmacognosy, and Research Institute of Pharmaceutical Sciences, School of Pharmacy, University of Mississippi, Oxford, MS 38677-1848, USA

**Keywords:** marine organisms, carbohydrate, glycoside, antioxidant, anticoagulant, anti-inflammatory, antitumor, antimicrobial

## Abstract

The oceans harbor a great diversity of organisms, and have been recognized as an important source of new compounds with nutritional and therapeutic potential. Among these compounds, carbohydrate-based compounds are of particular interest because they exhibit numerous biological functions associated with their chemical diversity. This gives rise to new substances for the development of bioactive products. Many are the known applications of substances with glycosidic domains obtained from marine species. This review covers the structural properties and the current findings on the antioxidant, anti-inflammatory, anticoagulant, antitumor and antimicrobial activities of medium and high molecular-weight carbohydrates or glycosylated compounds extracted from various marine organisms.

## 1. Introduction

The oceans cover about 70% of the earth’s surface, and harbor a great diversity of living beings, ranging from unicellular bacteria to large multicellular mammals [1]. The large biodiversity of the marine environment is also accompanied with great chemical variety, which makes this habitat a promising source of new biomedically active molecules [2,3]. Currently some products obtained from marine sources are in the clinical trials phase for possible use as analgesics [4], anticancer drugs [5], and for treatments against viruses [6,7,8]. Despite these studies, marine potential remains largely unknown.

Among the promising but poorly explored marine molecules are carbohydrates, which stand out for their varied structural and chemical characteristics. Besides participating in energy storage and as a structural component (especially in exoskeletons of invertebrates), carbohydrates also play many other key biological roles such as fertilization signaling [9,10,11], pathogen recognition [12], cellular interactions [13], tumor metastasis [14], in addition to important pharmacological activities such as antitumor [15,16], antiviral [17,18], anticoagulants [19], antioxidants [20] and anti-inflammatory [2,21,22].

In this review we will discuss about the structural and biological aspects of the various carbohydrate-based compounds of marine origin endowed with potential biomedical and biotechnological applications. The main goal of this report is to reinforce to the scientific community the great value of marine-derived carbohydrates and glycosylated compounds of medium and high molecular-weight (MW) to drug discovery and development. Although these molecules can present actions on multiple systems, attention is paid more to their antioxidant, anti-inflammatory, anticoagulant, antitumor and antimicrobial properties. 

## 2. Diversity of Carbohydrates from Marine Sources

Carbohydrates are the most abundant biomolecules on earth considering cellulose and chitin as the main representatives. These organic compounds act not only as the main energy source (as seen in starch and glycogen) but also as biologically functional structural players in events of cellular recognition, especially when present at the cell surface [23]. Carbohydrates are also the most complex biomolecules in terms of structure. The enhanced dynamic behavior, large conformational fluctuations, diversity of monomeric units accompanied by various enantiomers, multiple types of glycosidic bonds, and extensive post-polymerization modifications are factors that contribute to increase the structural complexity of these molecules. The carbohydrate classes are also vast. It can include both neutral and negatively charged saccharides with variable lengths. Famous examples are the *N*-linked or *O*-linked oligosaccharides in glycoproteins, glycosaminoglycan (GAG) in proteoglycans, glycolipids, sulfated fucans, sulfated galactans, among many other highly glycosylated products [24].

## 3. Structure and Function

### 3.1. Neutral and Acididc Polysaccharides

#### 3.1.1. Laminaran

**Figure 1:**
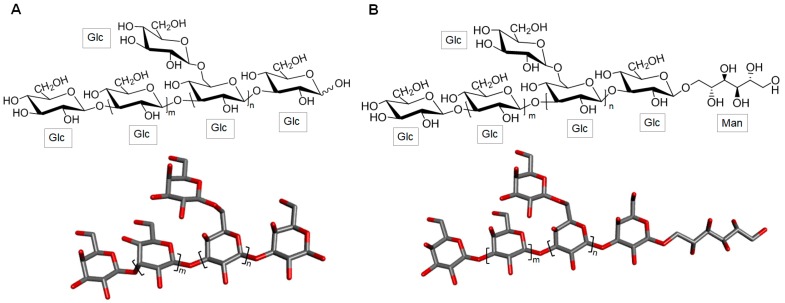
Representative chemical structure of laminaran which is composed of a backbone of 3-linked β-d-glucose (Glc) units with possible 6-linked branches of Glc residues and with reducing terminal ends with (**A**) Glc units (laminaran type G) or (**B**) D-mannitol (Man) residues (laminaran type M).

The main chain of laminarans, also found in brown alga, is mostly consisted of 3-linked β-d- glucose (Glc) residues (Figure 1) with a small proportion (usually less than 10%) of branches of single β-d-Glc residues attached to C-6 of the Glc residues of the backbone [25]. According to the reducing terminal ends, laminarans can be divided in two types: the first type with chains which are terminated by D-Glc residues (type G) (Figure 1A) and the second type with chains ending with D-mannitol (Man) residues (type M) (Figure 1B) [26]. The proportions of the two types of laminaran, and their consequent structures, vary according to the seaweed species. Environmental factors such as seasonal periods, salt concentration and frond age are additional influencing factors on chemical structures of laminaran [27]. Other environmental factors, including water temperature, salinity, waves, sea currents and depth of immersion (maybe pressure) have been also reported to influence on laminaran chemical composition [28]. Laminarans exist in either highly or poorly soluble forms. The first form is characterized by complete solubility in cold water, while the other is only soluble in hot water. The different solubility levels are influenced by the presence and number of branching residues. The higher the branching content, the greater the solubility in cold water [26].

Laminaran exerts many bioactivities such as anticancer, anti-inflammatory, anticoagulant and antioxidant effects [28]. A recent review was published discussing the anticancer effects of two brown algal polysaccharides and emphasis was given on laminaran [25]. In this review laminaran enhances the therapeutic effects of commercial anticancer drugs [25]. For instance, laminaran can inhibit the in vitro formation of colonies of colon cancer cells DLD-1. This polysaccharide also showed a synergistic effect with X-ray irradiation against this same cancer cell line by decreasing the amounts and size of the colonies [29]. In the study of Malyarenko et al., lamellar sulfates of *Fucus evanescens* showed the capacity to decrease the migration ability of cancer cells in vitro by inhibiting the activity of certain metalloproteinases such as MMP-2 and MMP-9 [30].

According to the publication of Lee et al., laminaran shows also the capacity to enhance the release of some inflammatory mediators [31]. This makes laminaran a potential therapeutic with immunostimulatory and anti-inflammatory properties [31]. With respect to antimicrobial activity, this marine glycan shows also the inhibitory capacity on both Gram-positive and Gram-negative bacteria such as *Salmonella typhimurium*, *Listeria monocytogenes* and *Vibrio parahaemolyticus* to adhere on HT-29-Luc cells of human enterocytes, besides inhibiting the invasion of *S. typhimurium* in this cell line [32]. The literature survey showed that laminaran is able to prevent HIV activity by decreasing (a) the adsorption of the HIV particle in human lymphocytes and (b) the efficiency of the HIV reverse transcriptase, which plays an important role in the proliferation of the virus during the infection cycle [33]. This study suggests that laminaran acts as an efficient inhibitor of HIV replication and proliferation [33].

#### 3.1.2. Alginic Acid

**Figure 2:**
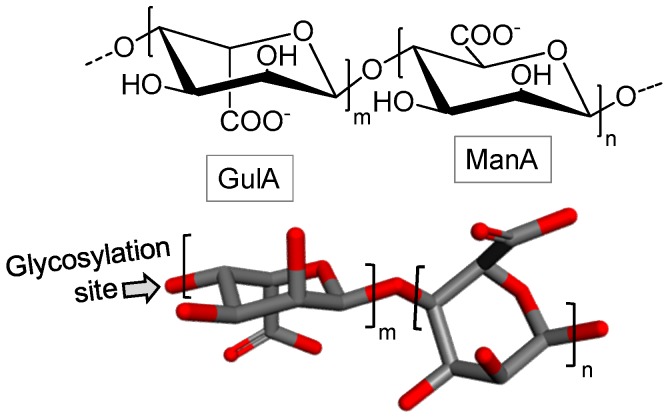
Chemical structure of alginate. It is composed of building blocks of α-l-guluronate (GulA) and β-d-mannuronate (ManA) units.

Alginic acid is a polysaccharide obtained from brown algae. It has linear structure and consists of β-d-manuronic acid (ManA) and α-l-guluronic acid (GulA) in repeating building blocks. These building blocks may be composed of consecutive GulA residues [GulA-GulA-GulA-GulA]_n_, consecutive ManA residues [ManA-ManA-ManA-ManA]_n_, or alternating ManA and GulA residues [GulA-ManA-GulA-ManA]_n_ (Figure 2) [34]. This polysaccharide has a wide spectrum of application in medicine, in the food industry, in biotechnology and in other industrial sectors [35].

In the study of So et al., alginic acid was shown to be a promising antioxidant agent against oxidative stress induced by free radicals [36]. In a work published five years later, Sarithakumari et al. investigated the antioxidant and the anti-inflammatory potential of alginic acid, isolated from the brown algal species *Sargassum wightii*, by in vivo assays using rats with induced arthritis [37]. Histopathological analysis of the animal paw tissue showed that treatment with alginic acid was able to decrease the paw edema as well as the inflammatory infiltrates in the studied animal models. This polysaccharide was also able to reduce the activity of various enzymes such as cyclooxygenase, lipoxygenase and myeloperoxidase, besides reducing the levels of C-reactive protein, ceruloplasmin and rheumatoid factor. Reduction of lipid peroxidation and increased antioxidant enzyme activity was also reported [37].

Supportively Endo et al. showed in a separate work two years later that alginic acid is able to eliminate free radicals and reduce the ferrous ion in stored pork [38]. The antioxidant activity of alginic acid was attributed to its capacity to chelate metal, to scavenge free radicals and to reduce ferric ions in the tissue. This last ability is quite useful in light of the elevated levels of ferrous ions in pork meat. The literature also reported the antimicrobial activity of this polysaccharide [38]. For instance, in the work of Neettoo et al., an alginate-based coating was tested in order to increase the microbiological safety in the digestions of cold-smoked salmon. This study demonstrated the efficacy of alginate to control the growth of *Listeria monocytogenes*, a bacterium responsible for serious infections, mainly those caused by salmon uptake [39].

### 3.2. Sulfated Polysaccarides

#### 3.2.1. Fucoidan

**Figure 3:**
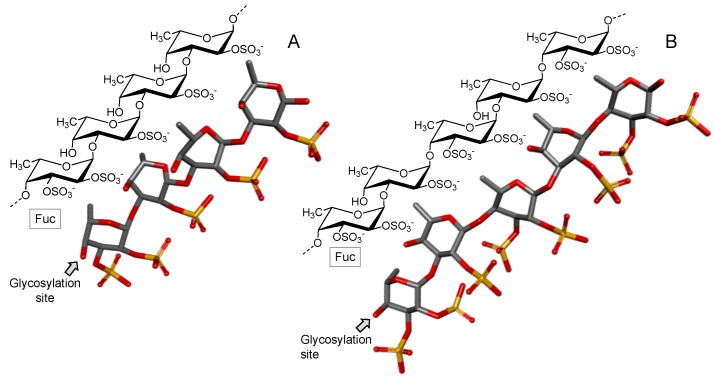
Fucoidans are polymers mostly composed of α-l-fucose (Fuc) residues either (**A**) mostly 3-linked or (**B**) 3- and 4-linked.

Composed of complex structure, fucoidans are obtained from brown algae. They generally consist of a backbone mostly 3-linked α-l-fucose (Fuc) (Figure 3A) or alternating α-l-Fuc residues with 3- and 4-glycosidic linkages (Figure 3B). Either case can be replaced with sulfate or acetyl groups, and/or side chains containing Fuc or other glycosyl units [40]. In addition to Fuc residues, they may contain small amounts of several other monosaccharides, such as Glc, galactose (Gal), xylose and/or mannose [41].

One of the first attempts to propose fucoidan structures was made in 1950 by Percival and Ross [42]. They analyzed the fucoidan-containg extract from *Fucus vesiculosus*. In order to understand some of the fucoidan’s biological activities, Patankar et al. have revised the fucoidan structure four decades later and described it as a polysaccharide consisted mainly of 3-linked α-l-Fuc units (Figure 3A) [43]. More recent papers stated that Fuc units in the fucoidan backbone can occur in the α-1,2 linkage type besides the α-1,3 and/or α-1,4 bonds [44]. It was also stated that sulfation can occur at C-2, C-3 and/or C-4 as well [44]. Despite the many published works regarding fucoidan, the relationship between structure and biological activities is not clearly and easily established because of the obstacles in full structure determination [45,46]. However, the scientific interest on fucoidan is so appealing because of the large spectrum of its application that intense research is annually carried out in terms of both structure and biomedical properties [47].

The highly cited review of Fitton covered potential applications of fucoidan in several types of therapies in which it was observed that the anti-inflammatory potential of fucoidan lies on its pleiotropic effects. These include selectin inhibition, complement inhibition and enzyme inhibitory activity [48]. In a comparative study of the anticoagulant property of fucoidans, extracted from various species of algae, *Laminaria saccharina* was found to furnish the fucoidan with the highest level of activity [49,50]. In vitro and in vivo assays are capable to evaluate the safety and clinical effects of fucoidan ingestion on homeostasis. Very strong in vitro anticoagulant activity has been observed as opposed to a modest effect on the in vivo assay [51]. Investigations on the antioxidant activity of fucoidan conclude that oral administration of this polysaccharide may lower serum parameters such as triacylglycerides, total cholesterol, low-density lipoprotein cholesterol and plasma Glc levels, and improve the anti-oxidation and innate immunity of catfish *Pelteobagrus fulvidraco* [52].

Other works have reported the anticancer activity of fucoidans extracts. An example is the well-cited paper from Cumashi et al. in which nine different fucoidans have been screened in terms of their multiple biomedical properties [49]. It has been shown that fucoidan from *L. saccharina*, *L. digitata*, *Fucus serratus*, *F. distichus* and *F. vesiculosus* have the capacity to block adhesion of MDA-MB-231 breast carcinoma cells, resulting in potential beneficial therapeutics against tumor metastasis [49]. Following the same rationale, other researchers have demonstrated that fucoidan of other seaweed species such as *Ecklonia cava*, *Sargassum hornery* and *Costaria costata* can present positive effects on human melanoma and colon cancer [53]. Fucoidan from other brown seaweeds like *Saccharina japonicus* and *Undaria pinnatifida* possess high antitumor activity and can inhibit proliferation and colony formation of breast cancer and melanoma cancer cell lines [54]. Fractions of native fucoidan and its derivatives have shown activity against the formation of colonies of two colorectal carcinoma cells, DLD-1 and HCT-116 [55].

The literature also mentions the antimicrobial properties of fucoidan [8,56]. An example of these is the publication of Thuy et al., where the anti-HIV activity of fucoidan, extracted from three brown algae *Sargassum mcclurei*, *S. polycystum* and *Turbinara ornate*, is reported [56]. All these fucoidan types tested in this work exhibited anti-HIV effects. The mechanism of action has been attributed to the capacity of this polysaccharide in blocking the first steps of HIV entry into the target cells [8,56]. A very recent study described the synthesis of silver nanoparticles (AgNPs) using fucoidan extracted from the alga *Padina tetrastromatica* as part of the coating material [57]. The focus of this work was on the increased antibacterial activity of antibiotics coated with AgNPs and fucoidan against antibiotic resistant bacteria. The synergistic effect of the combined antibiotics and the fucoidan in nanoparticles resulted in a two-fold increase of the antibacterial activity as compared to the antibiotic and the sulfated polysaccharide used in separate treatments.

#### 3.2.2. Carrageenan/Agaran

Carrageenans are sulfated galactans found in red seaweeds and composed of linear chains of alternating 3-linked β-d-Gal (conventionally ascribed as A units) and 4-linked α-d-Gal or α-d-3,6-anhydrogalactose (AnGal) (B units), thus forming disaccharide-repeating building blocks [58].

**Figure 4:**
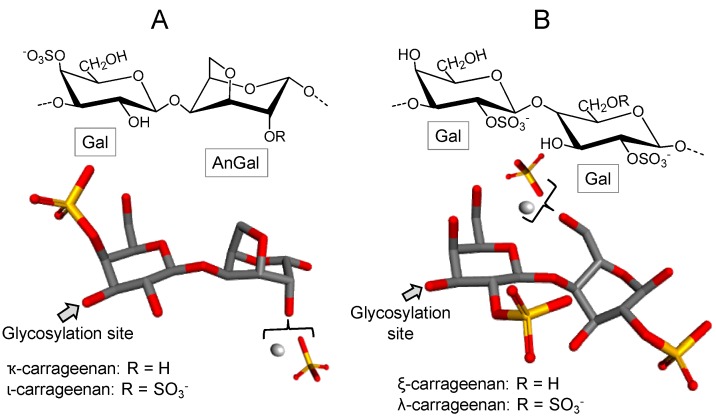
Representative chemical structures of carrageenans. These polymers are made up of alternating 3-linked β-d-galactose (Gal) units and (**A**) 4-linked α-d-anydrogalactose (AnGal) as seen in kappa (κ) and iota (ι) carrageenans or (**B**) α/β-d-Gal units as seen in zeta (ξ) and lambda (λ) carrageenans. These polymers also contain sulfate as their major substituent.

Carrageenans are classified according to the presence of the 3,6-anhydrous bridge at the 4-linked AnGal residues and the positions and numbers of sulfate groups (Figure 4). Carrageenans are traditionally identified by a Greek prefix accordingly to their structures. Structures vary in terms of sulfation patterns and the presence of AnGal units. The International Union of Pure and Applied Chemistry (IUPAC) establishes a nomenclature based on a code for the carrageenans: G = 3-linked β-d-Gal; D = 4-linked α-d-Gal; DA = 4-linked α-d-3,6-AnGal and S = sulfate ester (SO_3_^−^) [59,60].

In Figure 4, four illustrative structures are shown: (A) one composed of AnGal units, and (B) other composed of Gal units, both either in their sulfated or non-sulfated forms of occurrence. The three most commercially exploited carrageenans are kappa (κ), iota (ι) and lambda (λ). Their corresponding names, based on the IUPAC nomenclature and on the letter codes, are carrageenan 4-sulfate (DA-G4S), carrageenan 2,4-disulfate (DA2S-G4S), and carrageenan 2,6,2-trisulfate (D2S,6S-G2S), respectively [59,60]. In addition to these three major types of carrageenans, two other types, called carrageenans ѵ and μ are frequently found in carrageenan commercial samples. They are the biological precursors of ι- and κ-carrageenans, respectively [60].

In the food industry, carrageenans are widely explored because of their physicochemical properties like emulsifying, thickening, gelling and stabilizing effects. These properties give textural properties and protective effects to a wide range of food products [61]. Carrageenans are also widely used in the pharmaceutical and cosmetic industries [62].

Carrageenan-derived oligosaccharides produced by γ-irradiation exhibited antioxidant property in various assays such as the hydroxyl radical scavenging, the reduction power and the 1,1-diphenyl-2-picrylhydrazyl (DPPH) radical scavenging ability [63]. The effect observed was dose-dependent and the carrageenan types were also observed to have different impact on the antioxidant activity, following the order of λ < ι <κ [63].

Talarico et al. analyzed the action of λ and ι carrageenans against dengue virus serotypes. In this study, both carrageenans were shown to be potent inhibitors of the multiplication of dengue virus type 2 (DENV-2) and 3 (DENV-3) in Vero and HepG2 cells, with effective concentration values of 50% (EC_50_) of 0.14 to 4.1 μg [18].

Still, with respect to antiviral activity, Diogo et al. evaluated the action of λ-carrageenan against two viral pathogens of veterinary interest, bovine herpesvirus type 1 (BoHV-1) and suid herpesvirus type 1 (SuHV-1) [64]. λ-Carrageenan was able to reduce the infectivity of both types of virus. The concentration required to inactivate 50% of the virus, virucidal concentration (VC_50_) was 0.96 ± 0.08 μg/mL for BoHV-1 and 31.10 ± 2.28 μg/mL for SuHV-1. The antiviral activity of λ-carrageenan for BoHV-1, expressed in inhibitory concentration (IC_50_), was 0.52 ± 0.01 μg/mL, whereas for SuHV-1 was 10.42 ± 0.88 μg/mL.

In vitro tests have shown that ι-carrageenan is a potent inhibitor of the influenza A (H1N1) virus infection [65]. From this information Leibbrandt et al. decided to test a commercially available nasal spray containing ι-carrageenan in a model of influenza A infection in mice. Treatment of mice infected with a lethal dose of influenza A PR8/34 H1N1 virus and administered with ι-carrageenan at a concentration of 60 µg/mL repeated twice daily starting within 48 hours post-infection and resulted in strong protection of the mice, in a similar to those treated with oseltamivir [65].

Another study, also related to the antiviral potential of carrageenans, investigated the role of λ-carrageenan in the inhibition of rabies virus (RABV) infection [66]. The λ-carrageenan oligosaccharide (P32) specifically inhibited the replication of several RABV strains, and acted primarily by suppressing viral replication during the early post-adsorption period, thereby preventing viral internalization and viral fusion mediated by viral glycoproteins. The authors suggest that P32 derived from λ-carrageenan is a promising agent for the development of novel anti-RABV drugs [66].

Studies on the cytotoxic effects of κ- and λ-carrageenans on human cervical carcinoma cells (HeLa) and human umbilical vein endothelial cells (HUVEC) showed that both carrageenans had no significant effect on HUVEC (normal cells). However, both carrageenans were cytotoxic to HeLa, although λ-carrageenan has stronger cytotoxicity when compared to κ-carrageena. In addition, λ-carrageenan was shown to have a stronger effect on suppression of tumor cell proliferation and cell division compared to κ-carrageenan [67].

A study by a Russian group which investigated a κ/β-carrageenan extracted from the red alga *Tichocarpus crinitus* for its anti-inflammatory property through in vivo models. The work showed that the dose of 100 mg/kg of κ/β-carrageenan (orally administered) stimulates the induction of anti-inflammatory cytokines, such as interleukin (IL)-10, in mouse blood cells by more than 2.5-fold when compared to control. However, it has no effect on the production of pro-inflammatory cytokines, such as tumor necrosis factor-α (TNF-α). In addition, pre-treatment with carrageenan has been shown to reduce the excessive activation of the inflammatory cells triggered by lipopolysaccharide [68].

Murad et al. reported that the carrageenan (predominantly ι-carrageenan) extracted from the red alga inhibited cell growth, induced apoptosis and promoted DNA damage in a breast cancer cell line (MDA-MB-231) at the concentration of 50 μM [69].

Luo et al. reported the antitumor action of λ-carrageenan on melanoma cells B16-F10 and breast cancer 4T1. This carrageenan, when administered intratumorally at a dose of 50 mg/kg, can inhibit the growth of B16-F10 and 4T1 tumors in mice besides increasing the tumor immune response and increasing the number of immunostimulatory cell infiltrates as well as the production of pro-inflammatory cytokines. In addition, when used as a vaccine adjuvant, λ-carrageenan notably increased the preventive and therapeutic effects of vaccines against cancer [70].

In a recent work, McKim et al. investigated the effects of three commercial forms of carrageenan: λ, κ and ι. The parameters evaluated were intestinal absorption, pro-inflammatory signaling pathways, oxidative stress and cytotoxicity using four cell types (three of intestinal lineage, Caco-2, HT-29 HCT-8 and one hepatic lineage HepG2). None of the carrageenans showed any effect in the tests performed, and did not reproduce any of the in vitro findings already reported in the literature. At the conclusion of the study, the researchers reinforced the importance of further investigations to check reproducibility outside the discoveries of their laboratory, and before risk assessment, regimental decisions, or policy statements take place [71].

Agaran (and agarose) are structurally related polysaccharides of carrageenans. While carrageenans bear the β-d-Gal units in their backbones, agaran and agarose carry the α-l-Gal units. The chemical difference of agaran and agarose is that the latter is composed of the α-l-anGal unit [58]. These polysaccharides are also found in red seaweeds and can show different patterns of sulfation [58]. A recent paper of the group of Prof. Norma Benevides has shown the neuroprotective effects of a sulfated agaran isolated from the red alga *Gracilaria cornea* [72]. The study was developed on rats subjected to 6-hydroxydopamine (6-OHDA) in order to create an in vivo model of the Parkinson’s disease. The results have shown that 60 μg of sulfated agaran of *G. cornea* intrastriatally injected can promote neuroprotection in vivo as seen by reduction of the oxidative/nitroactive stress and by alterations of the monoamine contents promoted by 6-OHDA injection. In addition, this agaran was observed to also modulate the transcription of neuroprotection- and inflammation-related genes as well as returning behavioral activities and weight gain to normal conditions [72].

#### 3.2.3. Sulfated Polymannuronate

**Figure 5:**
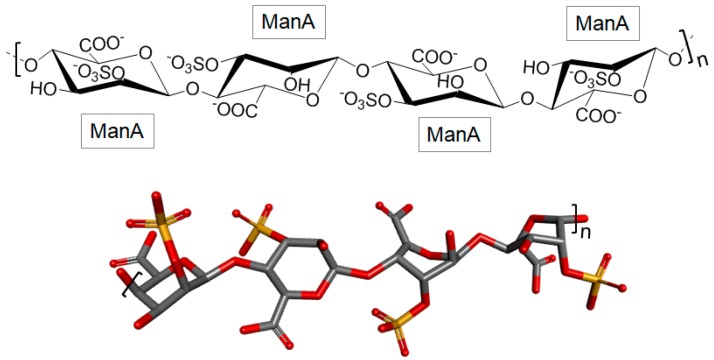
Structural representation of the brown algal sulfated polymannuronate (SPM). It is composed of 4-linked β-d-mannuronate (ManA) units in polymers with mean MW of 10 kDa. Sulfation can occur at either C-2 or C-3.

Sulfated polymannuronate (SPM), also known as sulfated polymannuroguluronate, is a sulfated polysaccharide extracted from brown algae, rich in 4-linked β-d-ManA with a mean MW of 10,000 Da (Figure 5) [73,74]. Sulfation can occur at either C-2 or C-3. The propylene glycol mannuronate sulfate is another version of this polysaccharide also with medical interest.

SPM has shown anti-HIV property [74]. SPM entered into the Phase II clinical trial in China, becoming the first marine sulfated polysaccharide with the potential to become a real anti-AIDS drug [75]. Several authors have focused on the elucidation of the molecular mechanism involved in the anti-HIV activity of SPM and their beneficial effects on the human cells of the immune system [75]. The particular study of Miao et al. has reported that CD4 is one of the possible targets for the specific binding of SPM on lymphocytes [75]. SPM-derived oligosaccharides have shown the capacity to interact, in multiple ways, with gp120, and therefore, present an anti-HIV outcome [74]. SPM can also inhibit the adhesion of the HIV Trans-activator of transcription (Tat) on SLK cells by direct binding to the KKR site (high-affinity heparin binding region) of Tat [73]. This structural information facilitates the elucidation of the structure-activity relationship of sulfated polysaccharides in the fight against HIV-1 infection.

#### 3.2.4. Glycosaminoglycans

Glycosaminoglycans (GAGs) are linear and heterogeneous sulfated glycans. Although structurally complex, the skeletons of these polysaccharides are simply constituted by repeated building blocks of disaccharides composed of alternating uronic acid (UroA) or Gal and hexosamine. The hexosamine may be glucosamine (GlcN) or *N*-acetylgalactosamine (GalNAc) and its differently substituted (mostly sulfated) derivatives. UroA can be either glucuronic acid (GlcA) or iduronic acid (IdoA) [76].

Heparin, heparan sulfate (HS), chondroitin sulfate (CS), dermatan sulfate (DS), keratan sulfate (KS) and hyaluronan (HA) are the major classes of GAGs found in animals. Although GAGs are all composed of repeating disaccharide units, the patterns of sulfation and the alternating monosaccharides that make up these units within the polymers vary significantly. The GAG classification is conventionally based on these structural variations. Interestingly, GAGs of marine organisms can present distinct structures from those from terrestrial animals, even considering the same class of GAGs [77]. Structural variations and heterogeneities of GAG chains (from either marine or terrestrial sources) especially in terms of sequence domains and the common occurrence in the extracellular matrix or on the surface of cells are all relevant contributing factors to the diversity of their biological and medicinal functions.

##### Heparin

**Figure 6:**
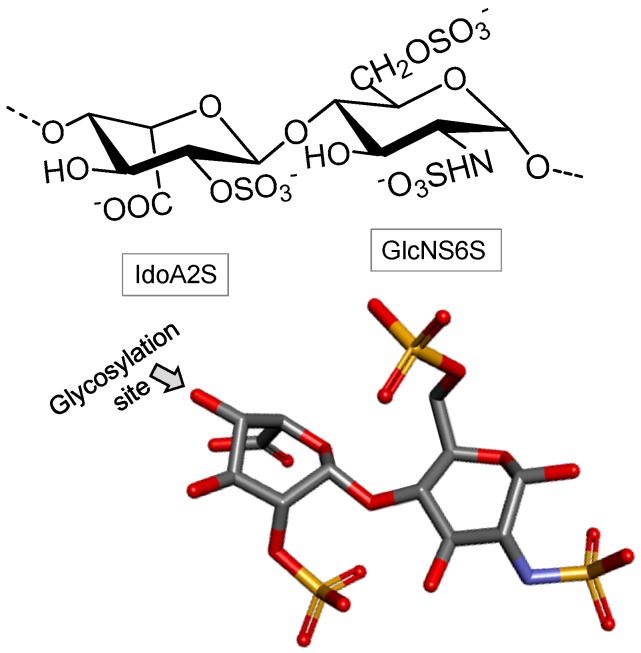
The heparin structure is mostly composed of alternating *N*,6-di-*O*-sulfated α- d-glucosamine (GlcNS6S) and 2-sulfated α-l-iduronate (IdoA2S) units, both 4-linked.

Heparin is mostly composed of alternating *N*,6-di-*O*-sulfated α-d-GlcN (GlcNS6S) and 2-sulfated α-l-IdoA units (IodA2S), both 4-linked (Figure 6). Among its occurrence in marine invertebrates, heparin is found in several phyla such as mollusks, crustaceans, annelids, echinoderms, tunicates and other urochordates [78]. In some of these invertebrates the heparin-like structures have presented structural peculiarities which are unique and not commonly found in the commonest and well-known mammalian-derived heparins. These unique properties may comprise low-levels of *N*- and 6-sulfation content and high-levels of *N*-acetylation on the GlcN units together with consistent amounts of GlcA units [79]. Naturally occurring low MW heparins are also found in marine invertebrates [80]. Works have also suggested that marine heparin structures are related to the species of occurrence and the chemical differences lie mostly on the relative abundance of the various composing disaccharide units or different chains [81]. In addition to these structural variations, the marine invertebrate heparin-like compounds also show variable biological functions [78].

Dietrich et al. reported the presence of a heparin in the crustacean *Penaeus brasiliensis* [80]. Of particular importance were the findings that this low MW heparin (LMWH) is enriched with non-sulfated UroA residues and exhibits potent antithrombotic activity. In vitro anticoagulant activity have shown that its effect is exerted on the inhibition of factor Xa and inhibition of thrombin (IIa) mediated mainly by cofactor heparin II (HCII) as opposed to mammalian heparins which exert their anticoagulant activity mainly through the inhibition of IIa and factor Xa mediated by antithrombin (AT). This shrimp-derived heparin has also presented potent in vivo antithrombotic activity as compared to the mammalian LMWH. Oppositely to the shrimp heparin, another heparin isolated from the crab *Goniopsis cruentata* has shown insignificant in vitro anticoagulant activity and low bleeding potency [78].

The heparin-like compound extracted from the shrimp *Litopenaeus vannamei* has shown capacity to reduce the influx of inflammatory cells in the lesion sites of a model of acute inflammation because this marine GAG is able to reduce the activity of the matrix metalloproteinases (MMPs) in the peritoneal lavage of inflamed animals [79]. This molecule has been reported to reduce almost 90% of the activity of MMP-9 secreted by activated human leukocytes besides presenting low hemorrhagic potential [79]. Another study has shown that this shrimp “heparinoid” is capable of suppressing the neovascularization process [82].

An analogue of heparin isolated from the ascidian *Styela plicata* was investigated in a model of colitis in rats [83]. The result observed was a decrease in the production of TNF-α, TGF-β and vascular endothelial growth factor (VEGF), as well as reduced activation of NF-β and mitogen-activated protein kinase (MAPK) kinase. At the cellular level, this tunicate heparin analogue can attenuate the recruitment of lymphocytes and macrophages and reduce apoptosis levels in epithelial cells. A drastic reduction in collagen-mediated fibrosis has also been observed [83].

##### Heparan Sulfate

Heparin and Heparan sulfate (HS) are structurally related GAGs since both are composed of GlcN units in their backbones, although with different concentrations [84]. HS is typically considered a less-modified heparin version. Among the sulfated GAGs, HS has the greatest structural variability. Depending on the tissue and species of origin, such a polysaccharide may be composed of several distinct disaccharide units, containing either GlcA or IdoA and GlcN with different extents of *N*- and/or 6-*O*-sulfation besides *N*-acetylation and 3-*O*-sulfation [84].

**Figure 7:**
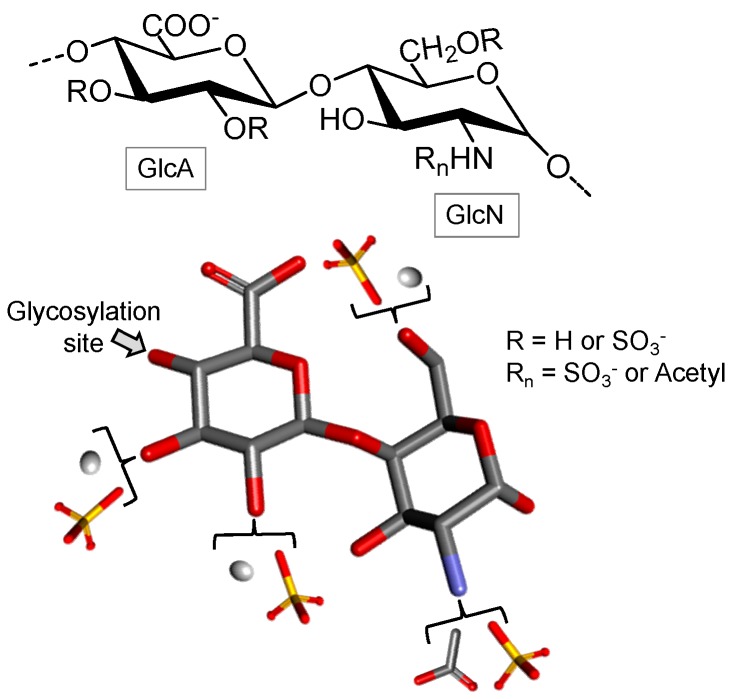
The heparan sulfate structure from the bivalve mollusk *Nodipecten nodosum* composed of alternating β-d-glucuronic acid (GlcA) and α-d-glucosamine (GlcN), both 4-linked. This molecule has a rare sulfation pattern on C-2 or C-3 of the GlcA units. The C-6 of GlcN can also be sulfated. The substituents of R_n_ can be either acetyl or sulfate.

For example, the HS isolated from shrimp *Artemia franciscana* possesses a high degree of *N*-sulfation and a relatively low degree of 6-*O*-sulfation of the GlcN residues. This compound exhibits high anticoagulant activity mediated by heparin cofactor II (HCII) [85]. In a study by Gomes et al., a novel HS structure with unique characteristics was isolated from the bivalve mollusk *Nodipecten nodosus* (Figure 7). This HS was reported to be formed by GlcA and GlcN units and by rare types of sulfation which can occur on C-2 or C-3 of the GlcA units [86]. This mollusk HS can inhibit thrombus growth without inducing the provoking hemorrhage. The same group reported later the action of this HS in inhibiting P-selectin-mediated events such as metastasis and recruitment of inflammatory cells [87].

##### Dermatan Sulfate

Dermatan sulfate (DS) is a linear variable-length polysaccharide composed of alternating disaccharide building blocks of 4-linked α-l-IdoA and 3-linked β-d-GalNAc units. These alternating disaccharide units can be variably sulfated at position C-2 of IdoA (IdoA2S) and/or C4 (GalNAc4S) and/or C6 (GalNAc6S) or both carbons (GalNAc4S6S) in the GalNAc unit, giving rise to different sulfated disaccharides [76].

**Figure 8:**
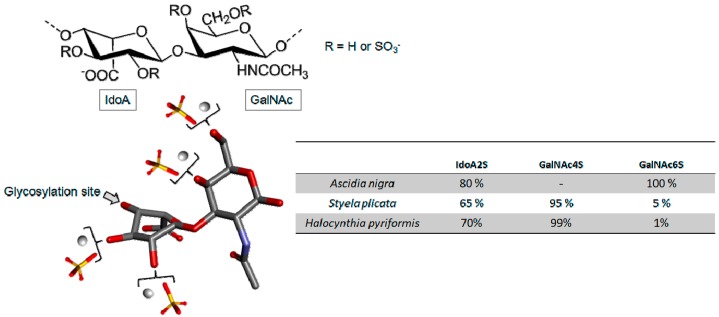
Representative chemical structure of dermatan sulfate (DS) which is composed of a backbone of 4-linked α-l-Idorunate (IdoA) and 3-linked β-d-*N*-acetylgalactosamine (GalNAc) units. The different radicals represent different patterns of sulfate substitutions. Ascidian DS are highly sulfated at C-2 of IdoA, but differ in the sulfation pattern at GalNAc. The insert table displays the sulfation rates of the ascidian species *Ascidia nigra*, *Styela plicata* and *Halocynthia pyriformis*.

In addition of being present in mammalian tissues, DS with high sulfation content was also found in different species of clam and tunicate [88,89,90]. The work of Pavão et al. [90] raised an interesting discussion about the structure-function relationship of DS, extracted from different species of ascidians (Figure 8). For example, DS isolated from *Ascidia nigra* is fully sulfated at C-6 of the GalNAc unit (100%) and at C-2 of IdoA (80%) (Figure 8). The DS from *S. plicata* however is less sulfated at C-2 (65%) and widely sulfated at the C-4 position of GalNAc (Figure 8). The DS, isolated from *Halocynthia pyriforms*, is similar to that of *S. plicata* (Figure 8).

Sulfation patterns in ascidian DS play a decisive role in biological actions. For instance, in anticoagulation the DS molecules from *S. plicata*, *H. pyriformis*, which bear more GalNAc4S units, exhibited a significant HCII-mediated IIa inhibition as opposed to *A. nigra* DS which did not show considerable anticoagulant activity [90,91]. With the exception of *A. nigra*, the two ascidian DSs displayed 10- and 6-fold more activity for HCII-related inhibition than the mammalian-derived native and oversulfated DS, respectively [90].

DS from *S. plicata* was also investigated regarding its anti-inflammatory activity in a model of colitis in rats [83]. This GAG exhibited a superior anti-inflammatory effect to that of mammalian heparin. This DS can decrease recruitment of lymphocytes and macrophages and apoptosis of epithelial cells. It is important to note that no hemorrhagic propensity has been pointed out after treatment with the ascidian glycan [83].

Kozlowski et al. investigated the effect of two DSs, isolated from *S. plicata* and *Phallusia nigra*, in the events of thrombosis, inflammation and metastasis [92]. The study showed that both GAGs can reduce thrombus size in a model of arterial thrombosis induced by FeCl_3_. In addition, they can also attenuate metastasis of MC-38 colon carcinoma, B16-BL6 melanoma cells and the infiltration of inflammatory cells in a mouse model of thioglycollate-induced peritonitis. The authors suggested that the observed effects are related to the inhibition of P-selectin [92].

##### Fucosylated Chondroitin Sulfate

**Figure 9:**
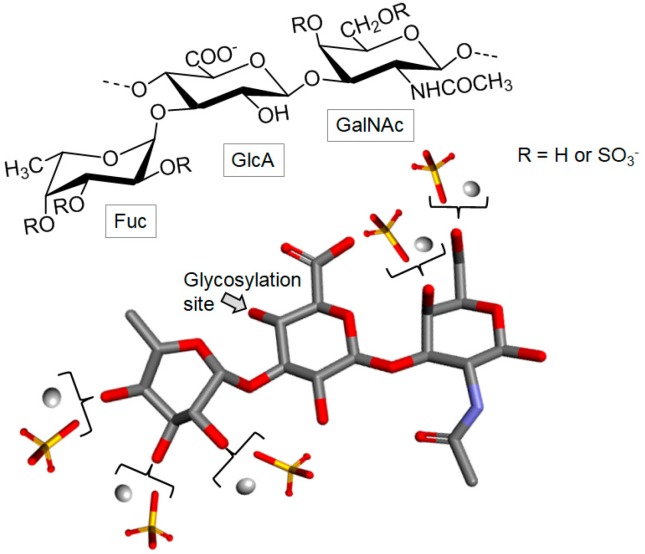
Structural representation of the holothurian fucosylated chondroitin sulfate (fCS). The structure is composed of α-l-fucose (Fuc), β-d-glucuronic acid (GlcA) and *N*-acetyl β-d-galactosamine (GalNAc).

Fucosylated chondroitin sulfate (fCS) is a distinct marine GAG found exclusively in sea cucumber (Echinodermata, Holothuroidea). This GAG is composed of the regular CS backbone with branches of α-l-Fuc units attached to C-3 of the GlcA residues (Figure 9). The lateral units of Fuc can show different patterns of sulfation according to the holothurian species [77,93].

With regard to the fCS’ therapeutic properties, this glycan exhibits a wide range of applications: anticoagulant [94], anti-metastasis [95] anti-inflammatory [96] and antiviral activities [97]. For this reason several papers have focused on the study of fCS. One of the works investigated samples of fCS extracted from three species of sea cucumbers: *Apostichopus japonicus*, *Stichopus chloronotus* and *Acaudina molpadioidea* in order to carry out a structural comparison among the three molecules and their antioxidant and anti-inflammatory properties. Analysis of ^1^H and ^13^C NMR of the polysaccharide identified three patterns of sulfation of the fucose branches: 4-*O*-, 2,4-di-*O*- and 3,4-di-*O*-sulfation. In addition, their activities were affected by the sulfation patterns of the Fuc branches, revealing that sulfation in O-4 is particularly important [98].

In the work of Ustyzhanina et al., the fCS isolated from the sea cucumber *Cucumaria japonica* inhibited platelet aggregation in vitro, and demonstrated significant anticoagulant activity. The latter activity was associated with the ability of fCS to increase the inhibition of IIa and factor Xa by AT as well to influence von Willebrand factor activity. The platelet aggregation inhibition process significantly distinguishes fCS from the LMWH [99]. fCS isolated from sea cucumber *Holothuria mexicana* exhibited high affinity with fibroblast growth factors 1 and 2. These factors are important in the neovascularization event. In addition, it displays the intrinsic anticoagulant activity and inhibited the activation of IIa and factor Xa by AT [100]. Still regarding anticoagulant property, a new fCS, isolated from the sea cucumber *Holothuria scabra*, was tested in comparison to heparin, and was shown to prolong activated partial thromboplastin time [101].

As mentioned above, the fucosylated sulfated polysaccharide also presents some antiviral properties, including against HIV. The anti-HIV action of holothurian fCS has generated a patent filed in the European patent bank [102]. Recent studies have reported the anti-HIV activity of the fCS obtained from the sea cucumber *Thelenota ananas*, which inhibited several strains of HIV-1 replication with different potencies. This study also reported that *T. ananas* fCS can bind potently to the recombinant HIV-1 gp120 protein, but did not inhibit recombinant HIV-1 reverse transcriptase [97].

#### 3.2.5. Propylene Glycol Alginate Sodium Sulfate

**Figure 10:**
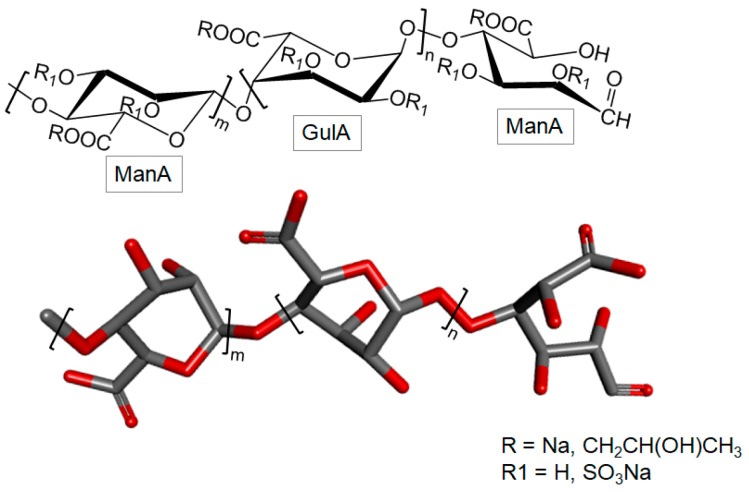
Structural representation of the propylene glycol alginate sodium sulfate (PSS).

Propylene glycol alginate sodium sulfate (PSS) is a sulfated polysaccharide, prepared by the chemical sulfation of the low MW alginate [103]. The backbone of this carbohydrate is composed of 4-linked β-d-ManA and 4-linked α-l-GulA, with degrees of sulfation substitution of 0.8 to 1.5 in the hydroxyls C-2 and C-3. It can also bear partially C-6-linked propylene glycol group at the hexuronic residues [104,105]. As a similar molecule to alginate, the blocks of these units constituting the PSS molecule may be composed of consecutive GulA residues [GulA-GulA-GulA-GulA]_n_, consecutive ManA units [ManA-ManA-ManA-ManA]_m_ residues or alternative residues of ManA and GulA [GulA-ManA-GulA-ManA]_n_ (Figure 10) [105].

With respect to biological activity, PSS has been used as a heparinoid drug to prevent and treat hyperlipidemia and ischemic cardio-cerebrovascular diseases for almost 30 years [103,106]. Because it has many important bioactivities, PSS has been the subject of many studies. This is proven by recent literature, where the various applications of this compound have been reported.

In the work of Xin et al. the anticoagulant and antithrombotic activities of low MW PSS were shown [104]. The low-MW PSS were prepared by oxidative-reductive depolymerization, and the activity tests were performed in vitro and in vivo. For the tests, four PPS fragments (FPs) with different MWs were used. The bioactivity evaluation showed a positive correlation between the MW and the anticoagulant and antithrombotic activities of the FPs. It was observed that FPs prolonged coagulation time and significantly reduced platelet aggregation. FPs also exerted an effect on factor IIa in the presence of AT and HCII, in addition to decreasing the weight and size of the thrombus in vivo [104].

Besides the anticoagulant activity, other PSS potentials have been explored. For example, Zhang et al. studied the anti-inflammatory potential of PSS through models of acute pancreatitis induced by Cerulein in mice. The results obtained by the authors indicated that PSS attenuates pancreas lesion by inhibiting autophagy and apoptosis through a mechanism involving the ratio between the MAPK/extracellular signal-regulated kinase (ERK). Inhibition of inflammatory factors, such as TNF-α, IL-6 and IL-1β, was also observed during the PSS-suppressed pancreatitis [103].

Also regarding the anti-inflammatory activity, Xu et al. investigated the protective function and mechanism of PSS in a hepatic ischemic reperfusion injury (RI) model in mice [106]. Pretreatment was performed at doses of 25 or 50 mg/kg, which were injected intraperitoneally into the animals 1 h 45 min prior to the induction of injury. The results indicated that pretreatment of PSS at both doses reduced the serum levels of the enzymes aspartate transaminase (AST) and alanine transaminase (ALT) compared to those observed in animals that did not receive pretreatment. PSS doses were also able to decrease the extent of hepatic necrosis, congestion and edema produced by IR injury. The authors suggested that pretreatment with PSS protected against hepatic IR injury [106].

More recent studies have sought a way to improve the bioavailability and efficacy of PSS in the body, since the absorption of this compound can sometimes be impaired because of its high MW. Faced with this challenge, Li et al. tested a new formulation of PSS in the form of enteric-coated nanoparticle (enteric PSS-NP) [107]. The study showed that the PSS nanoparticle had targeted intestinal absorption and improved pharmacodynamics. When compared to the PSS solution, enteric PSS-NP had an improved effectiveness in controlling body weight. These data indicate that enteric PSS-NP could be promising in terms of product development in the future [107].

### 3.3. N-acetylated Sugars

#### Chitin and Chitosan

**Figure 11:**
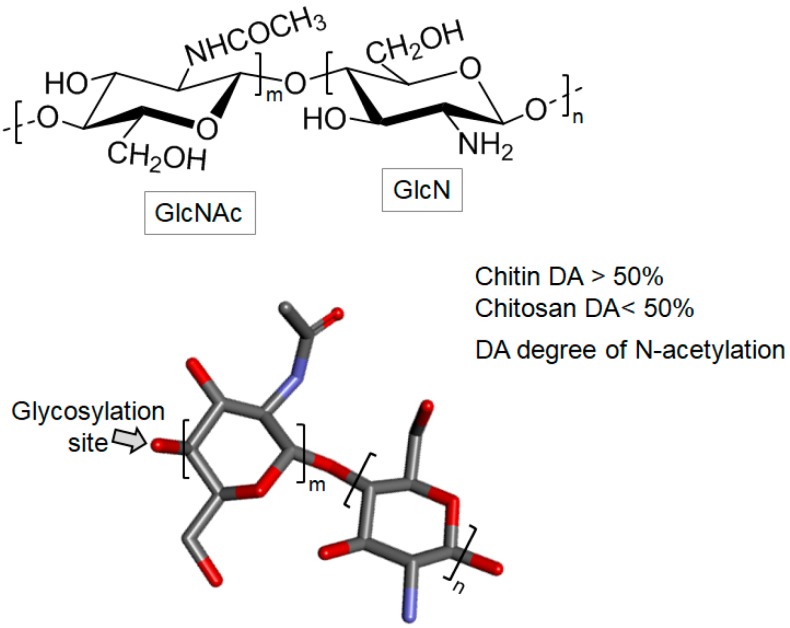
The chemical structures of chitin and chitosan. Chitin is consisting mainly of 2-acetamido-2-deoxy-d-β-glucose (*N*-acetylglucosamine, GlcNAc) units and partially of 2-amino-2-deoxy-β-d-glucose (glucosamine, GlcN) units, both 4-linked. When the degree of *N*-acetylation (DA) is less than 50% (GlcNAc content), the polymer is then named chitosan, otherwise, it is named chitin. DA is defined as the average number of *N*-acetylation per 100 monomers expressed as a percentage.

Chitin is an important constituent of the exoskeleton of many organisms such as crustaceans and insects. In the marine environment chitin is certainly the most abundant biopolymer, being structurally composed of GlcNAc and GlcN units bound by β-1,4 glycosidic bonds (Figure 11). In chitin, the GlcNAc content is higher than 70% of the total monosaccharide, making this polysaccharide highly *N*-acetylated. This, in turn, significantly decreases its water solubility property [24,108].

Chitosan is a cationic polysaccharide composed of the same units and the glycosidic linkage of chitin (Figure 11). However, low amounts of GlcNAc are found in chitosan, usually less than 50%. Physicochemical characteristics such as hydrophobicity and inter-chain interactions depend on the amount and distribution of the acetyl groups [24,108].

The chitosan molecule is non-toxic and has many biomedical applications, including bone tissue regeneration [109] and effects against a wide variety of pathogenic microorganisms [110,111,112]. Its proper use depends on many physicochemical factors and these factors can be managed accordingly to the levels of activity aimed for the chitosan. Examples of these factors are MW, degree of deacetylation, degree of substitution, length and position of a substituent in the GlcN units and pH [108].

The antineoplastic activity of chitin/chitosan and low MW chitin was evaluated using a human monocyte leukemia cell line, THP-1. Chitin and chitosan suppressed 100% growth of THP-1 tumor cells at concentrations equal to or greater than 1.5 mg/mL. The low MW chitin exhibited the same EC_50_ of 250 μg/mL [113].

Antioxidant property of chitosan was also investigated. Trung and Bao studied chitosan extracted from *L. vannamei* [114]. Their study suggested that the antioxidant effect observed was based on the free radical scavenging activity and the reduction potency. Another study related to the antioxidant effect of this marine glycan was carried out by Sarbon et al. [115]. In their work, the chitosan was extracted from the ladle shells species *Scylla olivacea*. The chitosan of *S. olivacea* exhibited a dose-dependent effect, where at the concentration of 10 mg/mL, the natural chitosan showed a greater reduction effect than the commercial chitosan.

Given the versatile applicability of this acetylated glycan, Divya et al. tested the antifungal and antioxidant activities of chitosan nanoparticles (ChNP) [116]. ChNP was tested in comparison to Amphotericin B, and showed good antifungal activity against all selected pathogens. The ChNP also exhibited significant antioxidant activity [116]. Previous work by the same group showed that chitosan nanoparticles inhibited the growth of clinically important microorganisms such as *Staphylococcus aureus*, *Pseudomona aeruginosa*, *Escherichia coli* and *Klebsiella pneumoniae* besides exhibiting antibiofilm activity with an inhibition rate of up to 98% [112].

A recent study was conducted with chitin/chitosan obtained from the shrimp shell *Penaeus monodon* [117]. These polysaccharides showed inhibitory effects on the proliferation of the human ovarian cancer cell line, PA-1. Chitin and chitosan can suppress 100% growth of PA-1 tumor cells at the respective concentrations of 50 μg/mL and 10 μg/mL, respectively [117].

### 3.4. Triterpene Glycosides

**Figure 12:**
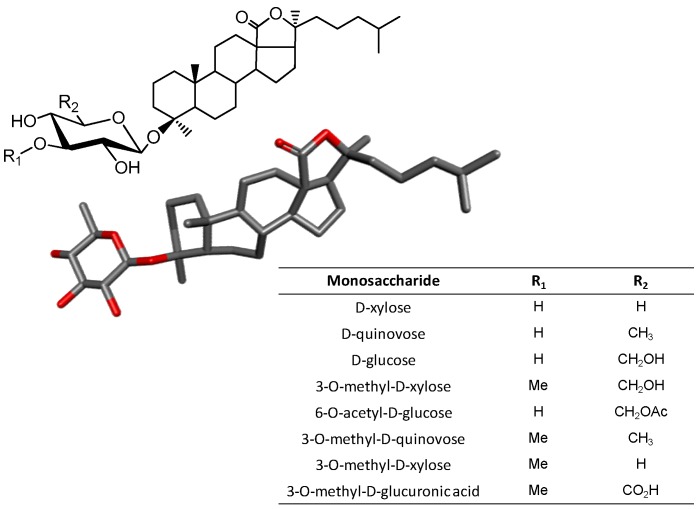
Chemical structures of glycosides with a triterpene backbone of holostane type bound to a sugar unit (glucose, Glc, in the case) and the possible substituents shown at the insert table.

The glycosides consist of amphiphilic compounds which contain a sugar bound to another functional group through a glycosidic bond (Figure 12). While the sugar can be a simple unit (monosaccharide) or various units (oligosaccharide) and the aglycone (functional group) may be a terpene, a flavonoid, or any other naturally occurring molecules [2,118].

Glycosides of marine organisms can be isolated from sea cucumber [119], starfish [120], sponge [121], alga [122] and coral [123]. Due to the great diversity of marine glycosides, many studies have focused on the investigation of their therapeutic properties. For example, glycosides isolated from the edible red seaweed *Laurencia undulata*, called floridoside or d-isofloridoside, have their antioxidant property investigated by Li et al. The two compounds showed significant antioxidant activity and are potential inhibitors of MMP-2 and MMP-9 [124].

Aurantoside K (a tetramic acid glycoside isolated from a sponge belonging to the genus *Melophlus*) exhibited a broad spectrum of antifungal activity against strains of *Candida albicans*, with the minimum inhibitory concentration (MIC) of 31.25 μg/mL for a strain resistant to Amphotericin B, and 1.95 μg/mL for a wild-type strain. It also showed a zone of inhibition of 14 mm of diameter in the concentration of 100 μg/disc for yeast *Cryptococcus neoformans*, 28 mm for *Aspergillus niger*, 31 mm *Penicillium* sp., 21 mm *Rhizopus sporangia* and 29 mm *Sordaria* sp. [125]. Another study carried out with a class of triterpene glycosides, called variegatusides, isolated from the sea cucumber *Stichopus variegatus* (Holothuriidae), and showed that these compounds have potent in vitro antifungal activity [126].

Wang et al. verified the cytotoxic effects of 13 purified triterpenic glycosides of *Holothuria scabra* and *Cucumaria frondosa* (Holothuriidae) against four human cell lines in order to advance the structure-activity relationship of these compounds [127]. The results showed that the number of glycosyl residues in the sugar chains and the aglycone side chain may affect their cytotoxicity to tumor cells and selective cytotoxicity in neoplastic versus normal cells. Works like this arouse interests in the use of these glycosides for the development of new antitumor drugs [115].

Given a vast number of actions that these compounds can have, it is worth understanding the underlying mechanisms by which these molecules act. A good option to uncover their molecular mechanisms of action of the marine glycosides is by identifying the relationships between their structures and activities. In a review of Park et al. the relationship between their effects and their structures were attempted on several molecular types. For example, stichoposide C and D, both isolated from the holothurian *Stichopus chloronotus*, exert anticancer activity [128]. However, the activity of the compounds occurs by distinct mechanisms due to differences in the sugar content. Stichoposide C has quinovose, and induces apoptosis through the generation of ceramide by the activation of acidic sphingomyelinase (SMase) and neutral SMase. Stichoposide D, which possesses Glc as the second monosaccharide unit, induces apoptosis by the activation of ceramide 6 synthase leading to the increase of cellular levels of ceramide.

Following the same reasoning, a recent study compared the effects of three frondosides (A, B and C) extracted from *C. frondosa* and its aglycone against pancreatic cancer cells. What can be observed was that frondoside A potentially inhibited the growth of pancreatic cancer cells with an EC_50_ of ~1 μM. Frondoside B was less potent with an EC_50_ of ~2.5 μM. Frondoside C and aglycone had no effect [129]. Frondoside A has potent antiproliferative, anti-invasive and antiangiogenic effects on a variety of cancers [130,131,132].

Cyclic steroid glycosides isolated from the starfish *Echinaster luzonicus*, namely as luzonicoside A (LuzA) and D (LuzD), were tested for their potential inhibitory capacity against RPMI-7951 and SK-Mel-28 melanoma cell lines. LuzA inhibited proliferation, colony formation and migration of SK-Mel-28 cells more significantly than LuzD. The authors suggested that their mechanisms of action are related to the regulation of the activity of cleaved caspase-3 and poly (ADP-ribose) polymerase (PARP), together with the levels of Survivin, Bcl-2, p21 and cyclin D1 [120].

### 3.5. Glycoproteins

**Figure 13:**
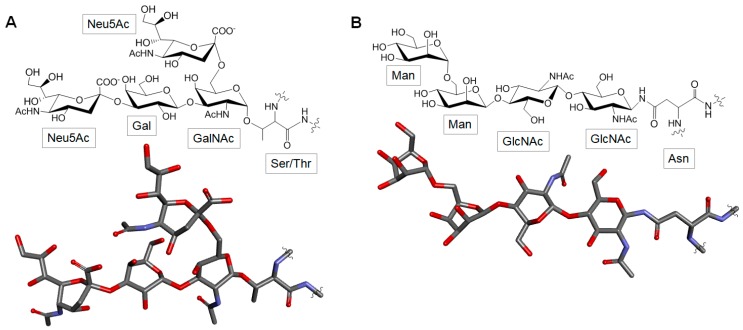
Chemical representatives of glycoproteins. While (**A**) *O*-linked glycans bind to the peptide chain by the hydroxyl group of a serine (Ser) or a threonine (Thr) residue, (**B**) *N-*linked glycans bind to the peptide chain by the amide group of an asparagine (Asn) residue.

Glycoproteins are glycoconjugates in which various monosaccharides are covalently attached to the protein backbone. Two major types of sugar chains (*N*- and *O*-linked) are found in glycoproteins. *N*-linked sugar chains contain a GlcNAc residue at its reducing end which is attached to the amide group of an asparagine (Asn) residue of the polypeptide backbone. The *O*-linked sugar chains contain a residue of GalNAc at its reducing terminus which is attached to the hydroxyl group of either a serine (Ser) or threonine (Thr) residue of a polypeptide backbone (Figure 13) [133].

Glycoproteins represent a large class of biomolecules. Many of the proteins that are components of cell membranes are glycosylated. Glycoproteins may have essential functions as receptors that capture various ligands into the cell such as transport proteins that are involved in the ingestion of various compounds, or as structures that mediate molecular recognition, signaling and interactions between cells [134]. For instance, lectins are excellent examples of biologically relevant glycoproteins found in marine organisms. Lectins are recognition proteins (glycosylated or not) of non-immune origin and endowed with the capacity to bind to the carbohydrate moieties of other glycoconjugates. Lectins play many varied biological functions including regulation of cell adhesion, recognition of molecules in cell-cell and cell-molecule interactions, and are also known to have vital immune functions [135]. Lectins are isolated from a variety of marine organisms including algae [136,137], sponges [138], mollusks [139] and echinoderms [140].

Many studies have reported the therapeutic effects of glycoproteins, especially lectins. For example, a study by Silva et al. aimed at the potential anti-inflammatory action of the lectin extracted from the red alga *Pterocladiella capillacea* [141]. The authors have observed a reasonable anti-inflammatory effect through both the paw edema model and the neutrophil migration model, based on the injection of carrageenan as an inflammation stimulus [141]. In a different work, the antinociceptive and anti-inflammatory effects of the lectin extracted from the red alga *Solieria filiformis* were evaluated [142]. In this work, the animals were pretreated with lectin by 30 min before receiving the nociceptive or inflammatory stimuli. The *S. filiformis* lectin significantly reduced the number of abdominal writhes and reduced the paw licking time in the formalin test. The lectin of *S. filiformis* also reduced neutrophil migration in a peritonitis model, in addition to reducing paw edema induced by carrageenan, dextran and serotonin [142]. In a recent work, Fontenelle et al. investigated the lectin extracted from the red seaweed *Bryothamnion triquetrum*, and reported its anti-inflammatory effect in mice [143].

Reports of anticancer activity of lectins have also been found in the literature. In one of the collected works, besides investigating the biological activity the authors also dealt with structural aspects of a lectin of the sea mollusk *Crenomytilus grayanus*. Cell viability assays have shown that *C. grayanus* lectin recognizes Gb3 globotriose on the surface of breast cancer cells, leading to cell death [144]. Also regarding anticancer activity, Liu et al. investigated the in vivo antitumor activity of hemocyanin (multifunctional glycoprotein) of the shrimp *L. vannamei* in Sarcoma-180 (S180) model of tumor-bearing mice [145]. After 8 days of treatment, the dose of 4 mg/kg significantly inhibited the growth of S180 to 49% compared to untreated animals [145].

In terms of antimicrobial activity, a new lectin was isolated from the green alga *Halimeda renschii*. The mannose-specificity lectin showed a potent activity against influenza virus in NCI-H292 cells at half maximal effective dose (ED_50_) of 2.45 nM. Antiviral action occurred through high affinity binding to hemagglutinin from the viral envelope [137].

### 3.6. Glycolipids

Glycolipids comprise a large and diverse group of lipids that serve numerous cellular functions [146]. They are amphipathic lipids, containing a hydrophilic portion composed of units of carbohydrates, from which gives its name (the prefix “glyco”). The lipid moiety is referred to as the hydrophobic tail, generally constituted of aliphatic fatty acid chains [147].

Among the classes of glycolipids are glycosphingolipids that are constituents of cell membranes in a wide variety of organisms (either from terrestrial or marine habitat) [148]. These compounds have biotechnological potential and play an important physiological role due to variations in their sugar chains. They are classified into cerebrosides, ceramide oligohexosides, globosides and gangliosides based on the constituent sugars (Table 1). In recent years, some glycosphingolipids have been isolated from marine invertebrates such as echinoderms, porifera and mollusks [149].

**Figure 14:**
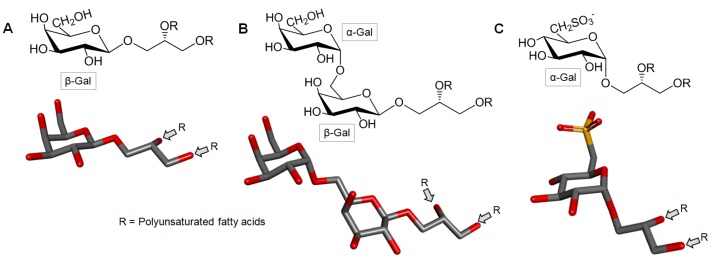
Representation of the general structure of the three main types of seaweed glycolipids. (**A**) Monogalactosil diglicerol (MGDG), (**B**) digalactosil diglicerol (DGDG) and (**C**) sulfonoquinovosyl dipalmitoyl glyceride (SQDG). R represents the acyl substituent chain.

Marine algae synthesize three major types of glycolipids, i.e., monogalactosil digliceride (MGDG), digalactosil diglyceride (DGDG) and sulfonoquinovosyl dipalmitoyl glyceride (SQDG) (Figure 14). These glycoglycerolipids are present in the chloroplasts of eukaryotic algae. MGDG and DGDG are the most abundant glycolipids of the thylakoid membrane and appear to play a crucial role in photosynthesis [150].

Many researchers have sought biologically active glycolipids from marine organisms to elucidate the structure-function relationships of glycolipids and to develop new medicinal resources. A good example of this was the study where eight new cerebrosides named Renierosides were isolated from an extract of the marine sponge *Haliclona* (*Reniera*) sp. The isolated compounds exhibited cytotoxicity of five human tumor cell lines, including human lung cancer (A549), human ovarian cancer (SK-OV-3), human skin cancer (SK-MEL-2), cancer cell line of the human central nervous system (XF498) and human colon cancer (HCT15) [151].

Plouguerné et al. identified SGDGs in fractions obtained after the purification of the organic extract of the *Sargassum vulgare* brown alga [152]. These metabolites exhibited antiviral activity against the herpes simplex virus type 1 (HSV1) and 2 (HSV2) viruses. The main SQDG responsible for anti-HSV1 and anti-HSV2 activities was characterized as 1,2-di-*O*-palmitoyl-3-*O*- (6-sulfo-α-d-quinovopyranosyl) glycerol [152]. Two SQDGs isolated from the red alga *Palmaria palmata* showed potent anti-inflammatory activity. Bioactive compounds were identified as (2S) -1-*O*-eicosapentaenoyl-2-*O*-myristoyl-3-*O*-(6-sulfo-α-d-quinazopyranosyl)-glycerol and (2S) -1-*O*-eicosapentaenoyl-2-*O*-palmitoyl-3-*O*-(6-sulfo-α-d-quinovopyranosyl-glycerol and showed nitric oxide inhibitory activity with IC_50_ values of 36.5 and 11.0 μM, respectively [153].

In the paper by Reyes et al. the first characterization of the MGDGs, DGDGs and glycosylceramides from *Isochrysis galbana* (Haptophyte) was described together with a study of their anti-inflammatory property as inhibitors of tumor necrosis factor α (TNF-α), a protein of cell signaling involved in the inflammatory response of the acute systemic phase [154]. In a recent paper, Che et al. have described that sea cucumber cerebrosides have improved learning and memory deficits, protecting against oxidative stress in vivo, and increasing the survival rate of PC12 cells, a rat pheochromocytoma cell line [148].

Overall, the bioactivities of the glycoglycerides are directly related to the sugar moiety. The position of the glycerol binding to the sugar, the length and the location of the acyl chain and the anomeric sugar configuration are all key structural contributors [155].

### 3.7. Iminosugar

Naturally occurring imino- or azasugars are monosaccharides whose oxygen heteroatom in the ring structure is replaced by nitrogen. In 1960 the first member of this class of compounds was isolated and characterized, a 5-amino-5-deoxyglucose antibiotic called nojirimycin. Subsequently, more than 25 additional nojirimycin analogs were described from plant and microbial sources [156,157].

**Figure 15:**
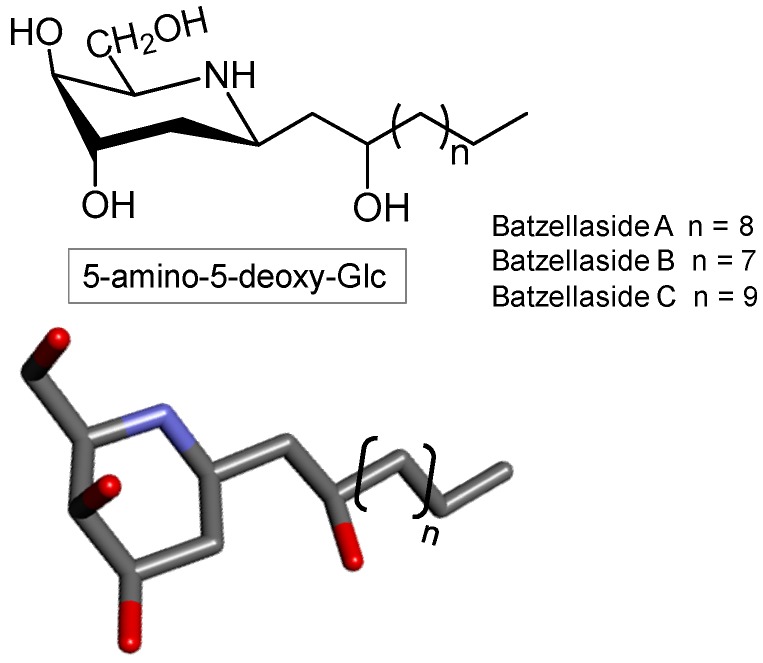
Representation of a nucleus of alkylated iminosugar. Batzellasides differ according to the length of the alkyl chain.

Iminosugars are commonly obtained from terrestrial sources or through chemical synthesis [158]. However, the work of Segraves and Crews described for the first time iminosugars from the marine environment [157]. In this work, three compounds were extracted from the sea sponge *Batzella sp*. and were presented as iminosugar nucleus with a long chain of alkyl substituent. They were identified as Batzellasides A, B and C (Figure 15). The identification of these compounds was made through comparison with the properties of known iminosugars derived from both natural and synthetic sources.

Iminosugars have potential therapeutic importance. These molecules can serve as antiviral [158], insecticidal [159], and nematicidal activities [160]. These potentials are associated with the ability of these molecules to selectively inhibit enzymes that degrade carbohydrates (glycosidases). The antiviral activity of iminosugars relies on its capacity to interfere with the glycoprotein processing [161].

Concerning the potential biomedical actions of iminosugars, the study of Segraves and Crews evaluated the antimicrobial action of the three iminosugars studied therein (batzellasides A, B and C) against the bacteria *Staphylococcus epidermidis* [157]. The three structures were able to inhibit the growth of this microorganism with MICs of 6.3 μg/mL [157].

The work of Sayce et al. has shown that the 1-deoxynojirimycin iminosugar bearing Glc and capable of inhibiting the production of infectious virus in vitro including dengue (DENV), hepatitis B, hepatitis C, HIV and influenza A viruses. Inhibition of endoplasmic reticulum α-glycosidases prevents virus release and is the main antiviral mechanism of action of iminosugars against DENV [161].

## 4. Concluding Remarks

The marine environment comprises a very rich source of biomedically potential compounds. Among these compounds, carbohydrate-based molecules and glycoconjugates are calling special attention, especially in light of the current development of the glycomics (sub) projects such as the marine medicinal glycomics [24]. Throughout this work we have seen a miscellany of investigations into the effects of marine glycans/glycoconjugate on human health. We have seen that in most of the time the beneficial effects of these molecules are related to their structural properties such as (a) length of the sugar chains, (b) monosaccharide composition, and above all, (c) the presence, (d) the type, and (e) the degree of substituents such as sulfation and/or acetylation. The chemical features of these molecules play a decisive role in their pharmacological properties. However, it is still necessary to understand the molecular mechanisms underlying these activities in order to understand the structural aspects of these molecules and what this diversity of structures may represent in the final effect. Despite advances in study techniques that have allowed a reasonable understanding of the structure-activity relationship and some underlying mechanisms of action of these compounds, clinical trials using marine glycans are still very scarce. At this point of the glycomics it is necessary to further evaluate the safety and efficacy of the carbohydrate-based molecules of marine origin, especially in the context of their large applications in potential formulation of new drugs and/or for the delivery of an end product to a specific site that a therapeutic intervention is required.

## Figures and Tables

**Table 1:** Classification of sphingolipids according to their sugar content.

Sugar Moiety	Glycosphingolipid
monosaccharide	cerebrosideo
disaccharide	ceramide dihexoside
oligosaccharide	ceramide oligohexoside
oligosaccharide + amino sugar	globoside
oligosaccharide + sulfate	sulfatide
oligosaccharide + sialic acid	ganglioside
